# Crowd-out of defence and health spending: is Israel different from other industrialised nations?

**DOI:** 10.1186/2045-4015-2-14

**Published:** 2013-04-22

**Authors:** Aaron Reeves, David Stuckler

**Affiliations:** 1 Department of Sociology, Manor Road, University of Oxford, Oxford, OX1 3UQ, UK; 2 Department of Public Health and Policy, London School of Hygiene & Tropical Medicine, 15-17 Tavistock Place, London, WC1H 9SH, UK

## Abstract

Does high defence spending limit the growth of public health investment? Using comparative data from 31 OECD countries between 1980 and 2010, we find little evidence that defence crowds out public health spending. Whether measured in terms of long-term levels or short-term changes, per capita defence and health spending positively and significantly correlate. To investigate the possibility that countries with high security needs such as Israel exhibit differing patterns, we also compare crowd-out among countries experiencing violent conflicts as well as current high military-spending countries. We observed a greater positive correlation between changes in health and defence spending among conflict-countries (r = 0.65, p < 0.01) than in non-conflict countries (r = 0.12, p = 0.01). However, similar to other high-military spending countries, Israel’s politicians reduced defence spending while increasing health expenditure during its recent recession. These analyses reveal that while Israel’s politicians have chronically underinvested in public health, there are modest steps being taken to rectify the country’s unique and avoidable crowding out of public health from its high military spending.

## Commentary

Does high defence spending limit the growth of public health investment? This notion, known as ‘crowd-out’, is theorised to apply in advanced industrialized nations [1,2]. Yet, it finds little support in empirical data [3]. Over the past three decades across OECD countries, there has been a significant and positive correlation between changes in defence spending and health spending (r = 0.16, p < 0.01, number of countries = 31) [4], even after adjusting for economic growth rates or using spending as a fraction of Gross Domestic Product, indicating that there is no inevitable crowding-out of public health from defence among high-income countries.

Hence, it is intriguing that in their paper, “Adjusting health expenditure for military spending and interest payment: Israel and the OECD countries”, Shmueli and Israeli, re-assess the priority Israel’s politicians place on public health starting with an implicit assumption that defence spending will crowd out public health investment [5]. They argue that Israel’s politicians allocate budgets “sequentially”. That is, “resources allocated to security and to paying the debt are determined first…and funding for all the other national needs is taken from the remaining*, primary civilian,* resources.”[italics in original]. Previously, this sequential principle was used to describe the Soviet Union’s allocation as that of ‘leftovers’: first the military took a large slice of the government budget, then the remaining limited funds were distributed among education, health, and social protection.

**Figure 1:**
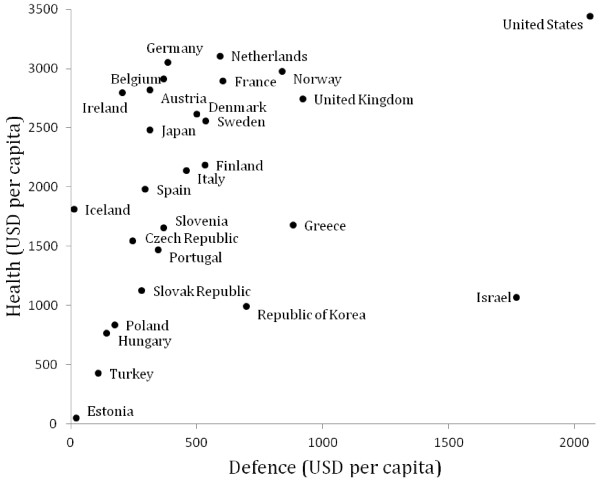
Defence and health spending in 2009, 27 OECD countries.

Unarguably, Israel’s budget choices are unique among industrialized nations. As shown in the scatterplot in Figure 1, health and defence spending are significantly and positively correlated. However, Israel is an outlier. It has low health but very high defence spending. About 17% of its government budget is allocated to the military. Once this huge strain on public budgets is removed, as the authors do, Israel’s public health budget as a fraction of government spending begins to converge with the median of industrialized nations.

Of course, each country has differing security needs, which has implications for their per capita spending on security. Yet, rather than critique Israel’s avoidable crowding out of public health from the militarization of the economy and government budgets, Shmueli & Israeli argue that it is necessary. The country has a high perceived security threat and high level of public debt, they argue, necessitating first covering these costs then allocating what remains as ‘primary civilian’ funds.

If the authors’ argument about sequential allocation, or ‘leftovers’, hypothesis were true for Israel, we would see two budget shifts. First, during Israel’s current recession, at a time when debt repayment rises and security needs have remained relatively constant (although some have argued these needs have increased in recent years), public health budgets would be reduced. Second, similar to Israel, we would see countries with high security needs, reflected in high military spending and incidents of violent conflict, exhibit a crowding out of defence budgets. To test these ideas, we used the Armed Conflict Dataset to measure conflict-years and -countries between 1980 and 2010 [6,7].

We found no support for either notion in the data describing Israel’s policy choices.

First, at a time of rising debt, Israel’s politicians chose to increase spending on health and reduce defence (see Additional file 1). It was a move that was triggered in part by popular protest. Street demonstrations calling for affordable housing, lower food prices, and more jobs and welfare spending occurred frequently throughout 2011 [8,9]. More than 400,000 people protested in Tel Aviv [10]. This social unrest led to the establishment of the Trajtenberg committee whose recommendations were partially implemented in October 2011, when the government voted to reduce military spending by $1.2 billion dollars, against vigorous opposition by the defence ministry [11-14].

Second, given Israel’s potential security risks, we compared Israel’s budget allocations with other countries with high security needs (as expressed by high military spending of >10% of public expenditure as well as experience of violent conflict). These countries included: Turkey, Korea, Chile, the United States, Azerbaijan, and Georgia. As shown in Table 1, we found that, similar to Israel, these high military spenders reduced defence spending between 2008 and 2010 *while* increasing health spending (except for the United States which had a large stimulus package and increased both defence and health) [15,16]. Additionally, we investigated countries which experienced significant conflicts, including Israel, Turkey, United Kingdom, and the United States from terrorist attacks and found a *greater* positive correlation between changes in health and defence spending in conflict-countries (r = 0.65, p < 0.01, number of countries = 4) than in non-conflict countries (r = 0.12, p = 0.01, number of countries = 29), indicating that in countries with high security needs health and defence are complementary (see Additional file 2: Table S1).

**Table 1:** Trends in public health and military spending among countries with high military spending (>10% of public expenditure), years 2008–2010

**Country**	**Military spending as a percentage of total public expenditure**	**Military spending as a percentage of GDP**	**Public health spending as a percentage of total public expenditure**	**Public health spending as a percentage of GDP**	**Real change in spending between 2008 and 2010**	
					**Military**	**Health**
Turkey	10.1	2.3	12.8	4.4	−0.7	120.5
Republic of Korea	13.6	2.8	11.9	3.6	−7.1	287.4
Israel	17.0	7.1	10.2	4.5	−153.8	113.3
Chile	18.0	3.3	15.6	3.3	−35.1	104.8
United States	18.8	4.4	19.4	7.6	148.4	642.1
Azerbaijan	22.1	3.3	3.1	0.8	−24.6	193.0
Georgia	29.3	8.5	4.8	1.8	−108.2	82.0

Source: World Bank World Development Indicators 2013 Edition.

Taken together, these budget choices in Israel and other industrialised nations reveal that health spending can crowd out defence spending, even in countries with high security needs, if policymakers choose to make public health a priority. In contrast with the argument in the accompanying paper, there is little empirical evidence to support a sequential allocation of defence followed by health budgets.

For public health policy in Israel, these observations have two important implications. First, in the short-term there is a need to recognise that the chronic underinvestment in public health is a problem in Israel. Israel stands out, even among high-military spenders, for failing to invest in public health systems. Recent budget shifts mark important steps towards rectifying the historically low priority placed on health, but there is still a long way to go to attain levels seen in other advanced industrialized nations. Second, in the long-term it will be important to recognise that health is a vital investment in security. Military spending may help protect against external threats (although this too is debatable given evidence that violence begets violence), but cannot substitute for investments that safeguard Israel’s most valuable asset: the health of its people.

## Competing interests

We declare that we have no conflicts of interest.

## Authors’ information

Aaron Reeves is a PhD candidate at ISER, University of Essex and a postdoctoral research associate at the University of Oxford.

David Stuckler, MPH, PhD, is a Senior Research Leader in Sociology at University of Oxford and research fellow of the London School of Hygiene and Tropical Medicine and Chatham House.

## Supplementary Material

Additional file 1: Figure S1 Health and Defence spending change between 2008 and 2010, 27 OECD countries. Click here for file

Additional file 2: Table S1 Association of changes in defence and health expenditures, 31 OECD countries, 1980-2010. Click here for file

## References

[B1] Deger S Military expenditure in Third World countries: the economic effects 1986 London: Routledge & Kegan Paul

[B2] Deger S Sen S Military expenditure: the political economy of international security 1990 Oxford: Oxford University Press

[B3] Kollias C Paleologou SM Budgetary trade-offs between defence, education and social spending in Greece Appl Econ Lett 2011 18 11 1071 1075 10.1080/13504851.2010.524606

[B4] OECD OECD.stat (database) Organisation for Economic Co-operation and Development Statistics 2011 OECD

[B5] Shmueli A Israeli A Adjusting health expenditure for military spending and interest payment: Israel and the OECD countries Isr J of Health Policy Res 2013 2 5 1 6 23425013 10.1186/2045-4015-2-5 PMC3583735

[B6] Gleditsch NP Wallensteen P Eriksson M Sollenberg M Strand H Armed conflict 1946–2001: a new dataset J Peace Res 2002 39 5 615 637 10.1177/0022343302039005007

[B7] Themnér L Wallensteen P Armed Conflict, 1946–2011 J Peace Res 2012 49 4 565 575 10.1177/0022343312452421

[B8] Pincus W United States needs to reevaluate its assistance to Israel Washington Post 2011 Washington: Washington Post Company

[B9] UPI Israel pressured to cut defense spending UPI 2011 London: United Press International

[B10] Kershner I Summer of Protest in Israel Peaks With 400,000 in City Streets New York Times 2011 New York: New York Times Company

[B11] UPI Israel: Generals say defense cuts perilous UPI 2011 London: United Press International

[B12] Bassok M Lis J Netanyahu strikes deal with Yisrael Beiteinu to approve Trajtenberg report Haaretz 2011 Tel Aviv, Israel: Amos Schocken

[B13] Post J Steinitz rejects budget criticism Jerusalem Post 2010 Jersualem: Jerusalem Post

[B14] Stoil RA IDF protests budget cuts, requests NIS 55 billion Jerusalem Post 2010 Jerusalem: Jerusalem Post

[B15] Mladovsky P Srivastava D Cylus J Karanikolos M Evetovits T Thomson S McKee M Health policy in the financial crisis Eurohealth 2012 18 1 3 6

[B16] Mladovsky P Srivastava D Cylus J Karanikolos M Evetovits T Thomson S McKee M Health policy responses to the financial crisis in Europe 2012 Copenhagen, Denmark: World Health Organization 2012 and World Health Organization, on behalf of the European Observatory on Health Systems and Policies

