# Supplementary material for: Crowd-out of defence and health spending: is Israel different from other industrialised nations?
Source: Isr J Health Policy Res. 2013 Apr 22;2:14. doi: 10.1186/2045-4015-2-14 (PMC3637214; doi:10.1186/2045-4015-2-14)
Supplement: Additional file 1: Figure S1 — Health and Defence spending change between 2008 and 2010, 27 OECD countries. [file 2045-4015-2-14-S1.doc]

Figure S1: Health and Defence spending change between 2008 and 2010, 27 OECD countries


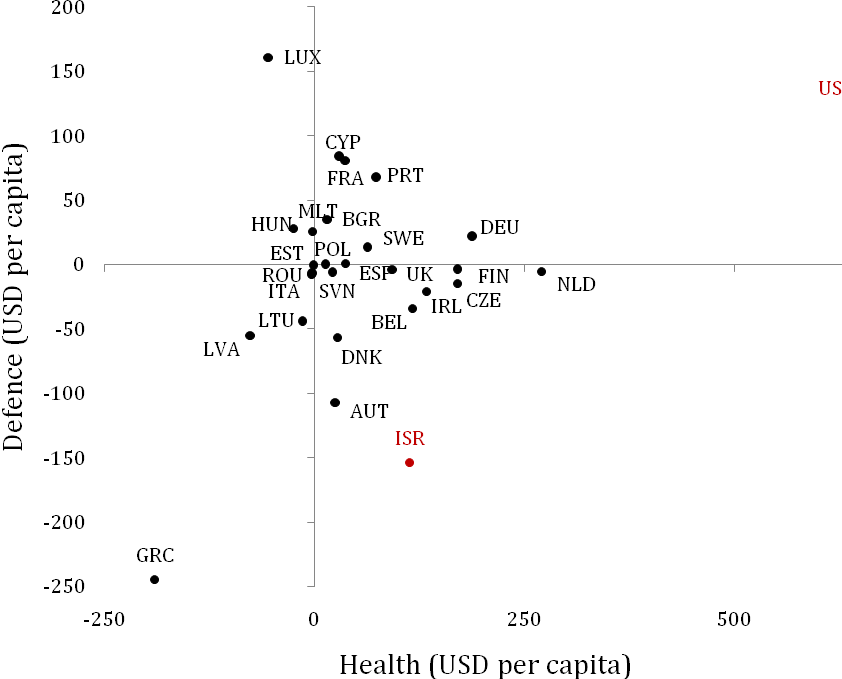


Notes: OECD Social Expenditure Database 2013 edition, EUROSTAT General government expenditure by function 2013 edition. Data are in constant 2005 USD adjusted for purchasing-power-parity.
