# Supplementary material for: Crowd-out of defence and health spending: is Israel different from other industrialised nations?
Source: Isr J Health Policy Res. 2013 Apr 22;2:14. doi: 10.1186/2045-4015-2-14 (PMC3637214; doi:10.1186/2045-4015-2-14)
Supplement: Additional file 2: Table S1 — Association of changes in defence and health expenditures, 31 OECD countries, 1980-2010. [file 2045-4015-2-14-S2.doc]

Table S1: Association of changes in defence and health expenditures, 31 OECD countries, 1980-2010

*Conflict Years and Non-Conflict Years*

|  | Total n = 31 countries | | Conflict n = 4 countries | | Non-conflict n = 29 countries | |
| --- | --- | --- | --- | --- | --- | --- |
|  | r | p-value | r | p-value | r | p-value |
| Association of health with defence spending | 0.16 | 0.00 | 0.65 | 0.00 | 0.12 | 0.01 |
| Adjusted for economic growth | 0.16 | 0.00 | 0.64 | 0.00 | 0.12 | 0.01 |
| Number of country-years | 453 | | 31 | | 422 | |

*Conflict and Non-Conflict Countries*

|  | Total n = 31 countries | | Conflict n = 5 countries | | Non-conflict n = 26 countries | |
| --- | --- | --- | --- | --- | --- | --- |
|  | r | p-value | r | p-value | r | p-value |
| Association of health with defence spending | 0.16 | 0.001 | 0.32 | 0.003 | 0.15 | 0.003 |
| Adjusted for economic growth | 0.16 | 0.001 | 0.32 | 0.005 | 0.15 | 0.003 |
| Number of country-years | 453 | | 80 | | 373 | |

*Notes:* Health and defence expenditure are in real purchasing-power-parity terms. Economic growth is adjusted for in percentage annual change. Conflict-years: Israel 1996-2009; Spain 1981-82, 1985-87, 1991-92; Turkey 2007-2009; UK 1991, 1998, 2003; US 1983, 1989, 2001-2001, 2004-2010.
